# Estimation of Engineering Properties for Coconut Shell Powder Reinforced 100% Bio-Degradable Beeswax Composites to Replace Wood Materials

**DOI:** 10.1016/j.mex.2025.103521

**Published:** 2025-07-18

**Authors:** Ramesh B T, Maruthi G V, Abhinav T, Shivakumar K Malladad, Lawrence J Fernandes, G M Mujeebullakhan, Ashok R Banagar, Srinivasa C V

**Affiliations:** a Department of Robotics and Automation, Symbiosis Institute of Technology (SIT), Symbiosis International (Deemed) University (SIU), Lavale, Pune, 412 115, Maharashtra, India; b Department of Mechanical Engineering, Global Academy of Technology, Rajarajeshwari nagar, Bangalore, 560 098, Karnataka, India; c Department of Mechanical Engineering, Dayananda Sagar Academy of Technology & Management, Kanakpura Road, Bangalore, 560 082, Karnataka, India; d Department of Mechanical Engineering, Sahyadri College of Engineering and Management (VTU, Belagavi), Adyar, Mangaluru, 575 007, Karnataka, India; e Department of Mechanical Engineering, STJ Institute of Technology, Ranebennur, 581 115, Karnataka, India; f Department of Mechanical Engineering, PES Institute of Technology and Management (Visvesvaraya Technological University, Belagavi), Shivamogga, 577 204, Karnataka, India; g Department of Engineering Design, GM University, Davanagere, 577 006, Karnataka, India

**Keywords:** Green composites, Beeswax, Synthesis, Coconut shell powder, Bio-Resin

## Abstract

Bio-degradability aligned with global warming will play a vital role to save the mother earth by saving the plenty of plants. Replacement of wood based structural parts with composites made with 100 % bio-degradable Bio-waste composites are the new trend in the structural stream now-a-days. Present work illustrates the synthesis of Beeswax to fabricate the composites in combination of coconut shell powder and preparation of composites of different grain sized coconut shell powder reinforcements. To understand the reason for improved mechanical properties SEM images were captured and a detailed analysis was made on these composites which shows the proper adhesion at 5000X magnification for 150 µm grain sized coconut shell powder reinforced composites. The following points will give the brief summary of the methods adopted,

• A method of bio resin extraction from beehives are being discussed in detail here in this method.
• Selection of proper grain sized bio-degradable reinforcements with proper proportions are being discussed.
• Mechanical and morphological analysis to understand the use of bio-degradable materials in the structural application and to replace the wood based materials.

This is one of the unique and new technique to make use of bio-degradable resin and reinforcements in the fabrication of green materials.

## Graphical abstract

**Figure:**
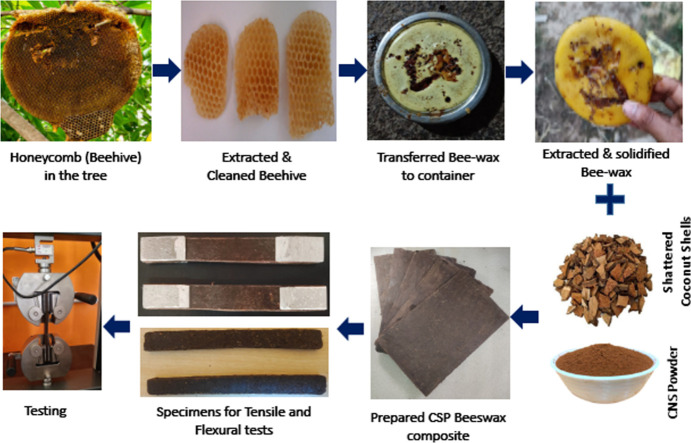

## Specifications table

This table provides general information on your method.

**Table:** 

Subject area	Engineering
More specific subject area	Bio-composites, Green Composites, Bio-Materials
Name of your method	Synthesis of Bio-resin from Beehive
Name and reference of original method	None
Resource availability	None

## Background

Few composite fabrication techniques have found applications across various industries such as automobile, aerospace, and construction, and has also made inroads into the textile and footwear sectors [1,2]. Agricultural biomass, along with waste from food, textiles, and paper pulp, has significant potential for creating biodegradable and flexible composites, as well as recyclable components for textiles and footwear [[3], [4], [5], [6], [7]]. Studies have highlighted the effectiveness of agricultural biomass sources such as corn and rice husks, banana leaves, sugarcane straw, pineapple leaves, and soybean straw as natural fiber reinforcements in biocomposites [[8], [9], [10]]. These bio-fillers enhance the biodegradability, cost-efficiency, and environmental sustainability of composite materials. This research aims to develop new nonwoven fibrous biocomposites by uniformly blending cellulose fibers derived from bacteria and plant leaves with polymers such as polycaprolactone (PCL), polylactic acid (PLA), and polyvinyl alcohol (PVA).

The primary focus of this study is on the development and characterization of bio-composite materials for potential future use in lightweight structural applications. To our knowledge, this is among the first studies to formulate 100 % bio-composite materials intended as substitutes for plywood, wood, or other structural materials.

Natural waxes such as beeswax have been widely utilized in packaging, coating, and barrier applications due to their hydrophobic nature and biodegradability. Previous studies have incorporated beeswax as a minor additive to polymer matrices (typically <10 wt%) to enhance moisture resistance and surface properties, primarily in thin films and coatings [11,12]. However, there exists no systematic investigation of beeswax as the primary structural filler in composite materials.

This study presents a novel approach wherein beeswax micro-particles of varying sizes (425 µm to 106 µm) are used as the primary filler in a beeswax-based composite matrix. While particle size effects are well-studied in synthetic fillers like diatomaceous earth or talc in polymers [13], their influence in natural wax systems remains unexplored. Furthermore, the dispersion behavior of these large-size micro-particles without sedimentation was investigated using optical microscopy and stability observations—filling a key gap in wax composite literature. Finally, this work provides a mechanical and density-based comparison with wood, which has not been addressed in earlier beeswax-based material studies. Together, these innovations offer a biodegradable structural material with properties potentially suitable as a wood alternative.

## Method details

In Bio composites are materials composed of natural fibers or particles, such as bamboo, hemp, flax or coconut, embedded in a polymer matrix. They offer a sustainable alternative to traditional composites, reducing reliance on non-renewable resources and lowering environmental impact. Bio composites are valued for their lightweight nature, biodegradability, and often comparable mechanical properties to synthetic composites, making them attractive for a range of applications from automotive and construction to consumer goods and packaging.

In recent years, there has been a growing demand for biodegradable composites reinforced with natural fillers sourced from plant fibers [14]. Fibers such as jute, flax, hemp, banana, sisal, and coconut provide excellent alternatives to synthetic fibers, reducing dependence on non-renewable resources and decreasing pollution and greenhouse gas emissions [15]. These natural fiber fillers offer several benefits, including low cost, high toughness, corrosion resistance, low density, good specific strength properties, reduced tool wear, and biodegradability.

Wood is prominent because of its remarkable heat resistance and non-melting properties. Wood, primarily composed of cellulose, lignin, and water, each with specific melting points, transforms under heat, producing various substances such as charcoal, methanol, and carbon dioxide. Lignin can be considered an effective environmentally friendly alternative to BPA, acting as a source of phenolic compounds. The content of these compounds is not readily predictable across various extraction processes, as indicated in the review by Lu and Gu [16]. Lignin is highly accessible and is the second most abundant organic material on Earth, following cellulose. It can be extracted from multiple sources, including wood, cotton, jute, hemp, and black liquor. Identifying the source and extraction method is essential, as it significantly impacts the structure and properties of the final product, as will be discussed subsequently. Lignin, owing to its abundance, sustainability, and polyphenolic structure, is considered a promising renewable raw material for bio-based epoxy resins [[17], [18], [19], [20], [21], [22], [23], [24]]. Lignin depolymerisation provides a pathway for producing alternative bio-based thermosetting systems, such as those derived from vanillin [[25], [26]]. Cellulose or lignin can also be used to derive resorcinol, a phenolic compound from which subsequent polymers are applied in adhesives, coatings, plastic mouldings, and rubber composite formulations. Additionally, different wood species such as oak, cedar, walnut, and certain mahoganies inherently possess tannic acid, a water-soluble polyphenolic compound characterized by a significant molecular weight. This compound serves as a bio-curing agent or epoxy monomer in epoxy systems [[27], [28], [29]], and it is also utilised as rosin, a significant and abundant natural product derived from conifer trees [[30], [31], [32], [33], [34]]. Also few researchers have made an attempt to study the acoustic emissions on the Kevlar and hemp based hybrid composites and also particle reinforced composites with glass fiber reinforced composites [[35], [36], [37]].

### Coconut shell and its powder as a filler

Coconut is a significant plantation and commercial crop in India, valued for its various applications, Typically, coconut shells, an agricultural residue, are disposed of by open burning, which releases toxic gases harmful to both human health and the environment. Known as "Kalpa Vriksha" in Sanskrit, meaning "the tree that provides all the fundamental needs of human life," the coconut palm contributes over 830,000 lakh rupees to India's GDP and about 6 % to the edible coconut oil market. Additionally, the coconut industry generates foreign exchange earnings of around 130,000 lakh rupees annually through the export of coconuts and coconut byproducts. Coconut also supplies raw materials for various industries such as oil milling and coir production. There is significant potential for products like shell charcoal, shell powder, and coconut milk powder [16].

Coconut shell powder (CNS) is a non-edible solid food waste with potential applications that can help reduce the use of synthetic fibers. CNS is a low-cost, lightweight material that can lower production costs and transportation fuel consumption. This study focuses on the incorporation of CNS into various matrices, emphasizing the mechanical properties of CNS composites.

Fig. 1

**Fig. 1:**
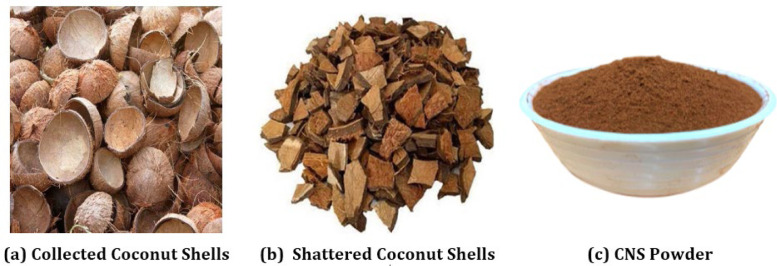
Processing of Coconut shells (CNS).

### Beeswax as resin

Natural resins are organic substances secreted by plants and trees, often in response to injury or as a protective measure against insects and pathogens. Natural resins have been used for thousands of years for various purposes, including as adhesives, coatings, vanishes, and in traditional medicine. They offer advantages such as biodegradability, renewable sourcing, and sometimes unique aesthetic properties, making them valuable in a wide range of applications across industries like woodworking.

Beeswax is a valuable byproduct that can generate significant income in addition to honey. Interestingly, one kilogram of beeswax is more valuable than one kilogram of honey. Unlike honey, beeswax is not a food product, making it easier to handle since it does not require meticulous packaging, thereby simplifying storage and transport. However, the potential of beeswax as a source of income is often overlooked in some tropical regions. In certain African countries like Ethiopia and Angola, where traditional fixed comb beekeeping is common, beeswax is a significant export. Conversely, in other areas, beeswax is often discarded. Many honey hunters and beekeepers worldwide are unaware that beeswax can be sold or used to produce high-value local products. There is often a lack of knowledge about the value of beeswax and its processing methods. Although precise statistics are unavailable, it is estimated that only about half of the global beeswax production reaches the market, with the remainder being discarded and wasted.

Bee wax is a natural resin produced by honeybees. It's used in various applications, including art, cosmetics, and woodworking, due to its adhesive and protective properties. As a resin, it offers durability and a pleasant scent, making it popular in many industries. By using the coconut shell powder and the bee wax as a resin the green composite that is biodegradable composite is prepared in the present study by considering the environmental safety.

### Binder/Hardener

Xanthan gum (XG) is a natural, water-soluble, bio-based powder characterized by its double helix structure, making it an effective binder in composite materials. XG can be utilized as a binder for cork, where oxidation enhances its water resistance. Additionally, XG serves as a binder for cellulose fibers in composite materials, with the cellulose fibers reinforcing the XG network. For the present work, xanthan gum was sourced from the online platform (Amazon).

### Synthesis of beeswax and sample preparation

To extract and purify beeswax from honeycombs, start by removing the honey and washing the combs thoroughly. Cut the cleaned combs into small pieces and place them in a pan, adding clean water to cover the combs. Heat the mixture gently, stirring continuously to avoid igniting the highly flammable wax. Once the combs are fully melted, pour the mixture into a long, tightly secured bag made from sacking, woven rush, nylon, jute, or a similar heavy fabric. Squeeze the bag over a basin or bucket using two pieces of wood to extract the wax, leaving behind larger debris such as brood, wood, and grass. Allow the bucket with the hot water and molten wax to cool in a clean, sheltered, and cool location. As the wax cools and solidifies, it will form a disc on the water's surface, while any remaining particles will settle at the bottom. After cooling completely, remove the wax layer and scrape off any residual material on its underside. *Re*-melt the wax with an equal volume of clean water and strain it through a finer cotton cloth to remove smaller impurities. Collect the filtered hot wax and water mixture in a lightly soaped enamel bowl, ensuring the bowl holds no more than two kilograms of wax. Avoid using fat or heavily scented soap, which could contaminate the wax. After cooling for approximately 12 h in a dust- and wind-free area, the beeswax can be easily removed from the bowl and any impurities on the bottom scraped off with a sharp knife.

After the extraction of Beeswax, measured quantity (measurement with respect to weight fraction of constituents) of Beeswax and CNS powders (of varying grain size from 425, 300, 212, 150 and 106 µm) were mixed thoroughly in a hot pan and 1 to 2 % of xanthan gum is added to this mixture to initiate the hardening process of this mixture. Micron-sized filler fractions (425, 300, 212, 150, and 106 µm) were obtained via a two-step top-down process. First, the raw material was mechanically milled (e.g., ball or roller mill), imparting impact and shear forces to fracture particles. Then, the milled powder was passed through a vibratory sieve stack configured with standard sieves matching the target micron sizes. Each fraction was collected between mesh apertures—e.g., particles retained between the 300 µm and 212 µm sieves correspond to the “212 µm” fraction. Iterative milling and sieving ensured narrow size distributions per standard granulometry norms (ISO 3310–1:2016 and ASTM E11–23). Once these constituents were mixed thoroughly, then the mixture is transferred to a mould and it will be allowed for cooling for 24 h.

As per the ASTM standards (ASTM-d-3039-M08 and ASTM-790-M) CNS reinforced Beeswax composites were prepared for tensile and flexural tests with the sample dimensions 200 × 20 × 5 (mm) (*L* × *B* × *T*) and 120 × 12 × 5 (mm) (*L* × *B* × *T*) respectively. To ensure uniform dispersion of filler in the beeswax matrix, the composite was prepared by high-shear mixing at 70 °C for 4 min at 12,000 rpm. Glycerol and cellulose nanofibers were added to increase the viscosity and maintain dispersion stability during cooling. This technique follows the methodology reported by Li et al. (2023) [38], where TEMPO-oxidized cellulose nanofibers and glycerol stabilized beeswax-water emulsions and prevented phase separation. Microscopic analysis confirmed the uniform distribution of fillers in the matrix without sedimentation [38].

Fig. 2

**Fig. 2:**
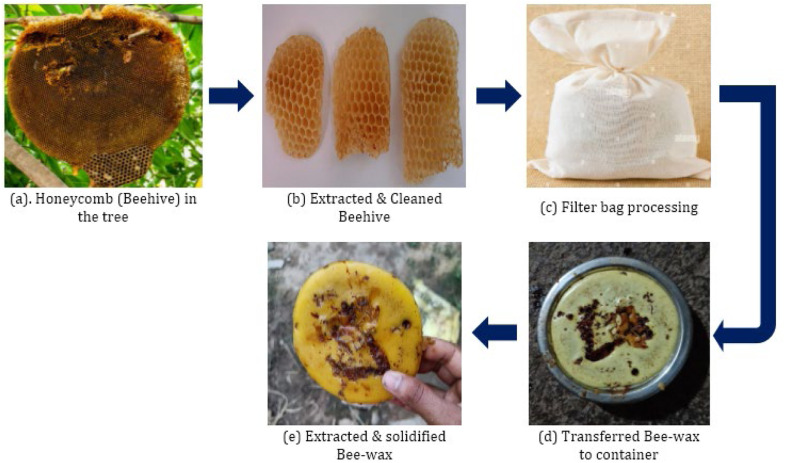
Various stages of Beeswax synthesis.

To validate the composite structure and determine the volume fractions of filler and matrix, the density of each sample was measured following ASTM D792 using the Archimedes method (buoyancy in water). Density values were then compared with theoretical densities computed via rule-of-mixtures to assess dispersion quality and detect void content. This analysis enabled estimation of filler loading, porosity, and verification of material homogeneity, consistent with composite characterization practices in the literature

Fig. 3

**Fig. 3:**
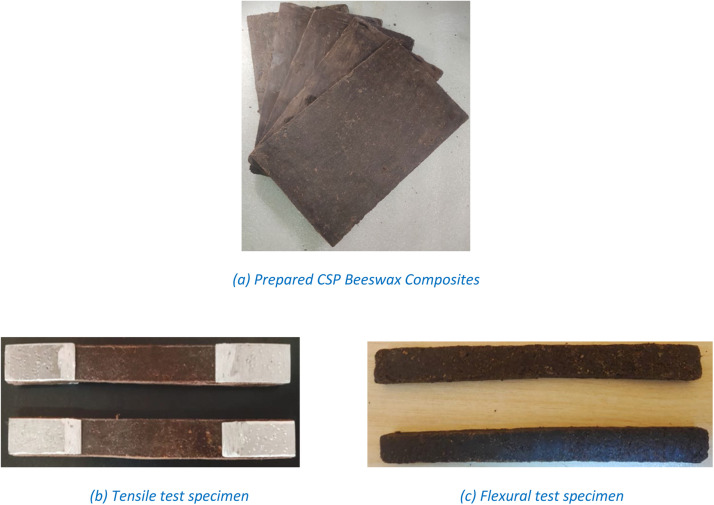
Fabricated CNS Reinforced composites (as per ASTM).

### Testing

#### Mechanical characterization

A small scale, Advanced Equipment’s made universal testing machine with the 10 kN capacity was used to examine the composite samples for tensile and flexural tests. Total of 5 replicates were prepared and all the samples were tested in UTM and average of 5 samples are reported in this study. Following table will depict the code adopted for the samples prepared for various mechanical test in the present work.

Table 1

**Table 1:** Code described for the samples prepared in the present work.

Code for samples prepared	CNS Powder grain size in microns	Sample code for tensile test	Sample code for flexural test	Sample code for SEM Analysis	
				Sample code	Magnification*
GS4	425	GST4	GSF4	GS4	5000X
GS5	300	GST5	GSF5	GS5	
GS6	212	GST6	GSF6	GS6	
GS7	150	GST7	GSF7	GS7	
GS8	106	GST8	GSF8	GS8	

⁎ Magnification was kept common for all the sample codes.

#### Morphological study

A scanning electron microscope (SEM) is a sophisticated electron microscope that employs a focused beam of electrons to create highly detailed, magnified images of a sample's surface. This technique relies on the interaction between the electrons and the atoms in the sample, generating signals that provide insights into the sample's composition and surface topography. SEMs are capable of achieving magnifications up to 3000,000 times and can image materials with a resolution of only a few nanometers.

The SEM testing at NMIT College, Bangalore, utilizes a Zeiss EVO 10 model. The specifications of this model include a tungsten filament and a magnification range from 7X to over 100,000X. It can accommodate specimens with a maximum height of 100 mm and a diameter of 230 mm. The motorized stage travel in the XYZ axes measures 80 × 100 × 35 mm. The microscope operates in High Vacuum (HV) mode, Variable Pressure (VP) mode, and Extended Pressure (EP) mode. It features an Element EDS system, an optional backscatter detector, and the Smart SEM Touch for intuitive operation. Additionally, the device employs a lanthanum hexaboride (LaB6) electron emitter.

## Method validation

### Tensile test

**Fig. 4:**
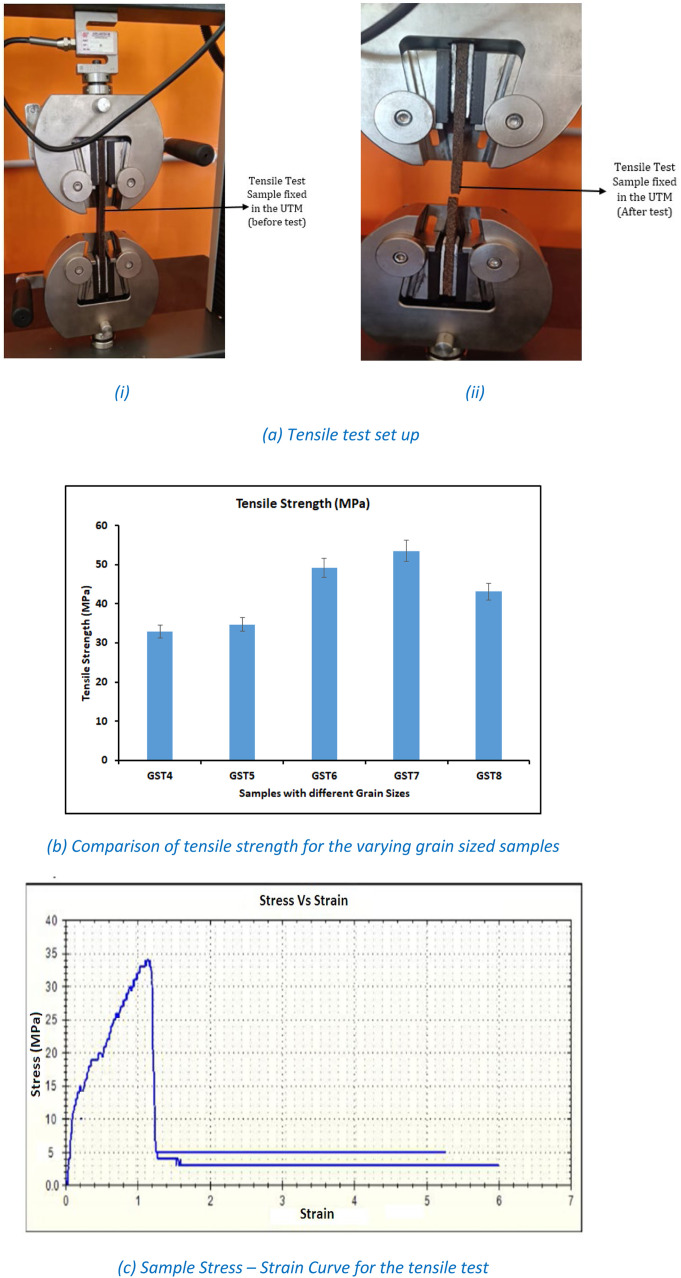
Tensile Test details for CNS-Beeswax composites.

Fig. 4(a) depicts the arrangements made to conduct the tensile test on CNS fibers reinforced Beeswax composites. Fig. 4(a) consists of two sections which shows the tensile test sample fixed in the UTM set up before and after the tensile test. After the tensile test, results were computed for five compositions of CNS reinforced Beeswax composite. Similarly, Fig. 4(b) shows the tensile strength values obtained for the various grain sized composites. Through this Fig. 4(b) it is concluded that, among five compositions composites made with 150 µm grain sized CNS reinforcements (GST7) have exhibited the highest tensile strength of 53.54 MPa as compared to all other grain size combinations. Composite made with higher grain sized CNS reinforced composites (GST4) have shown poor tensile strength of 32.95 MPa. This variation in the peak load for smaller grain sized composites is due to the close bonds developed between grains of CNS powder and Beeswax. As the grain size reduced, complete Beeswax encirculation was possible for all the grains which has improved the bonds between the adjacent grains. This has improved the bond strength and resulted in the enhanced peak load. Similarly, for the higher grain size the phenomenon was quite reversible and the bonds developed between the larger grains has resulted in the poor strength the thus composites prepared with larger sized grains has shown poor tensile strength.

### Flexural test

**Fig. 5:**
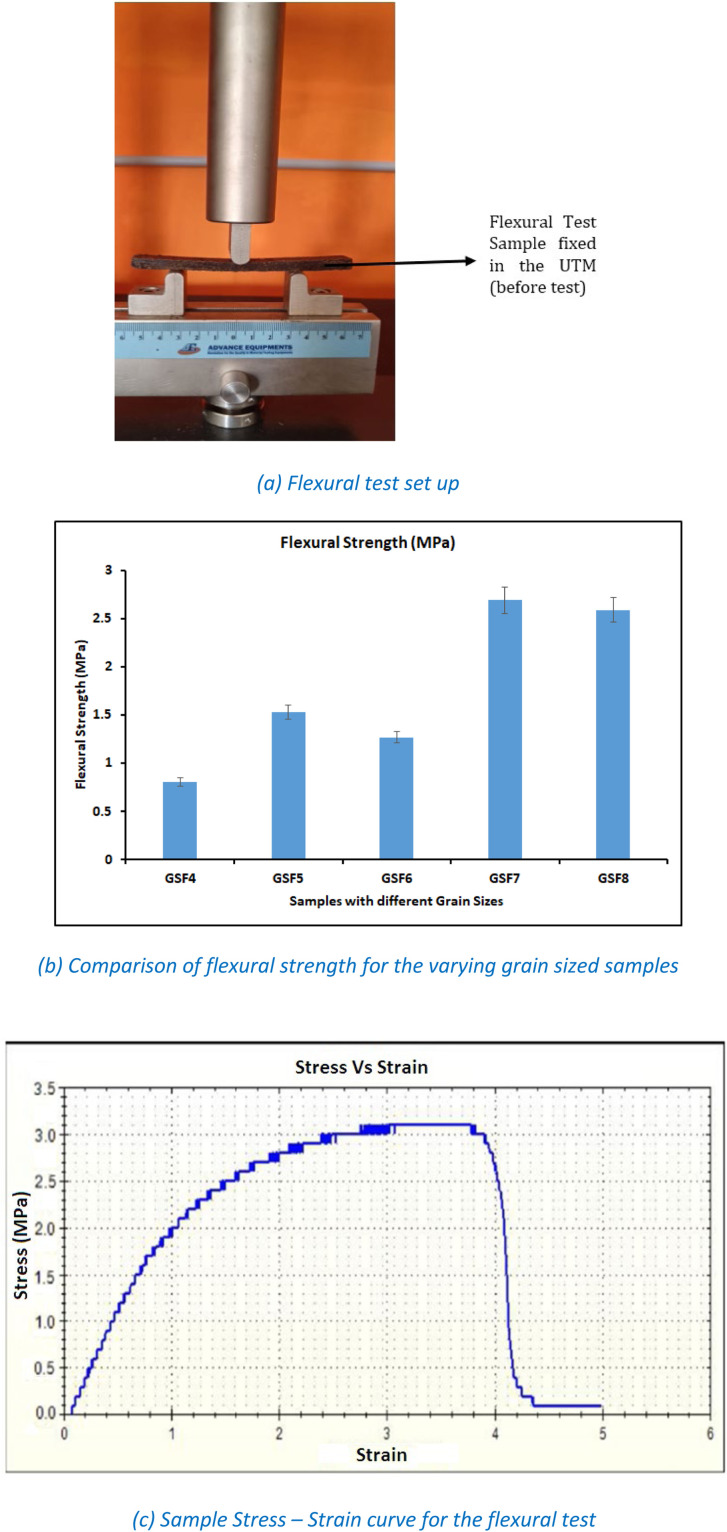
Flexural Test details for CNS-Beeswax composites.

Fig. 5(a) depicts the arrangements made to conduct the flexural test on CNS fibers reinforced Beeswax composites. In this figure, composite sample was tested under three-point bending set up. Similarly, Fig. 5(b) shows the comparison between the various grain sized beeswax composites. Through this Fig. 5(b) it is concluded that, among five compositions composites made with 150 µm grain sized CNS reinforcements have exhibited the highest flexural strength of 2.69 MPa as compared to all other grain size combinations. Composite made with higher grain sized CNS reinforced composites have shown poor flexural strength of 0.8 MPa. Similar to the mechanism of tensile samples, the variation of flexural load was addressed. The reason for the variation in the flexural load is due to the poor and strong bond strength between the higher and lower grain sized CNS powder reinforced CNS composites.

### Morphological analysis

**Fig. 6:**
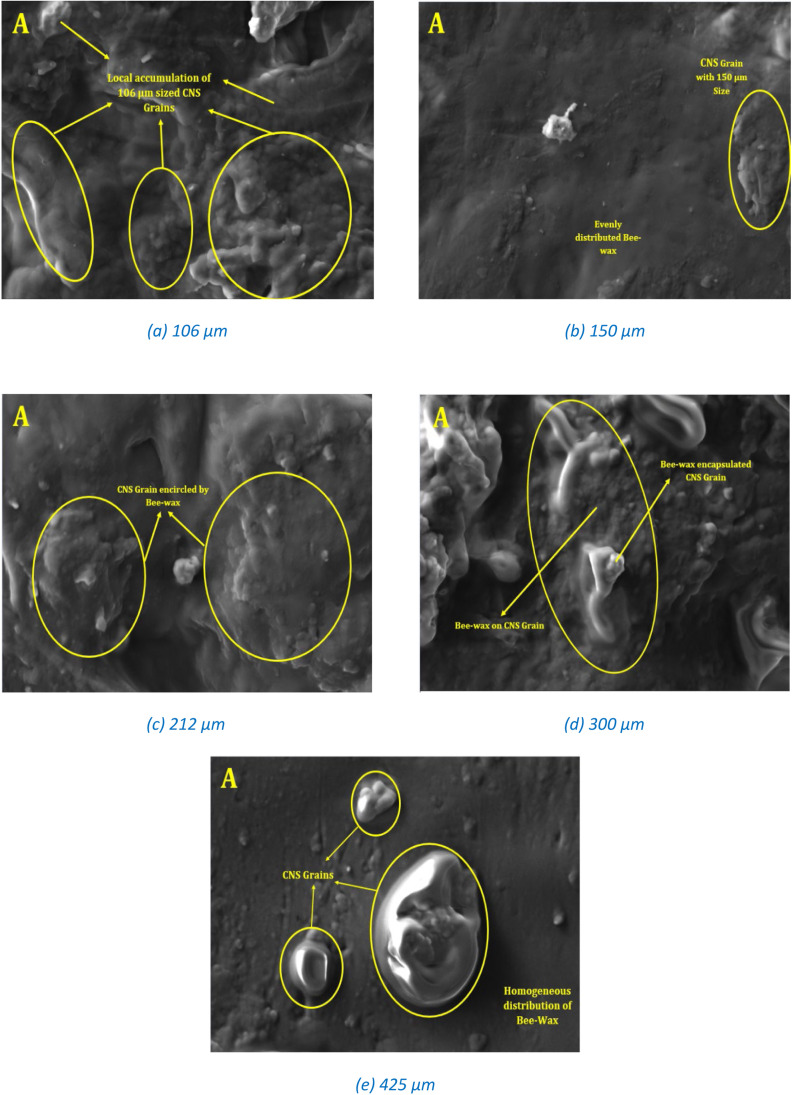
SEM Images of CNS powder reinforced Beeswax composite.

Figs. 6 illustrate the surface topography, porosity, and interfacial adhesion between the filler particles and the beeswax matrix. SEM analyses were conducted on samples GS4, GS5, GS6, GS7, and GS8, and the results were compared at different magnifications including 5000X. The surface topography of the fabricated composites reveals variations in mechanical properties by providing phase information of the composite specimens.

Fig. 6 depicts the SEM images for the different grain sized (Fig. 6(a) through (e) represented with grain sizes 106, 150, 212, 300 and 425 µm respectively) CNS powder reinforced Beeswax composites. Among which Fig. 6(a) shows the local accumulation of CNS powder in the fabricated composite due to the smaller grain sizes. Fig. 6(c) and (d) shows the entrapped air cavity between Beeswax enclosed CNS grains. And Fig. 6(e) shows the homogeneously distributed Beeswax in which CNS grains were pulled out due to the higher surface area of contact, due to which they have shown reduced mechanical properties as compared to other combination of grain sizes. Fig. 6(b) depicts the composite made with 150 µm grain sized CNS powder reinforced composites. It shows the neatly distribute CNS powder in the evenly distributed Beeswax and due to which the results for this category samples were high as compared to all other grain sized composites. It will help Beeswax to produce the fiber encapsulation and results in the continuous chain to produce the stronger bonds. Similarly, as the grain size increases, CNS fibers were freely suspended in the Beeswax resin which is creating the dis-continuous chains and that will have reduced the strength of the composite. After analyzing the SEM images for all the categories of the grain sizes in the present work, it is observed that as the grain size decreases that results in the enhanced mechanical properties.

Microscopic analysis confirmed uniform distribution of beeswax microparticles across all size ranges in the composite matrix, with no visible sedimentation during sample preparation or curing (Fig. 6). The absence of phase separation indicates a stable interface between the filler and matrix—contrary to common concerns regarding sedimentation of micron-sized particles in low-viscosity systems [3].

Mechanical characterization results, including tensile and flexural strength, showed a clear dependency on particle size, with optimal performance observed at 150 µm particle size (Fig. 6(b)). Importantly, the composite's mechanical and density values were benchmarked against those of commonly used wood materials. The comparison reveals that beeswax-based composites with optimized filler sizing can achieve performance comparable to softwood or engineered wood, presenting a promising alternative for biodegradable structural applications [2].

This work introduces a novel class of biodegradable structural composites utilizing beeswax microparticles as the primary filler material, an approach not previously reported. The study fills three critical research gaps:

1. Particle size-controlled composite design using beeswax across a micron range;
2. Quantified proof of uniform filler dispersion within the matrix, overcoming sedimentation challenges; and
3. Mechanical performance benchmarking with wood, targeting real-world structural use.

These outcomes establish the foundation for using natural wax composites in sustainable material design for future wood-replacement applications.

## Limitations

Use of these composite boards could be done for low load applications where we can replace wooden boards. A comparison table between wooden materials with present Bio-based composites were depicted in the Table 2 below. This describes the use of bio resin based natural composites uses in the place of wood materials. From the below table it can be seen that, the flexural strength of the composites discussed in the present work is very low and thats due to the particulate form of composites fabrication and thus it has led to the reduced flexural strenght of the composites.

**Table 2:** Mechanical Properties of various Wood materials [[39], [40], [41]].

Sl. No.	Type of Wood Materials	Tensile Strength (MPa)	Flexural Strength (MPa)	Reference(s)
1	Douglas Fir	2.34	84.12	[39]
2	White Oak	5.51	104.80	
3	Wester N Red Cedar	1.52	51.71	
4	Sitka Spruce	2.55	70.33	
5	Mahogany	–	79.29	
6	Teak	–	100.66	
7	Pine	115.2	–	[40]
8	Particle Board	10.89	–	[41]
9	Hard Board	37.58	–	
10	PP + 40 % Wood Flour	52.3	72.4	
11	Present Study (Bio-Based Composites)	53.54	2.69	

## Ethics statements

In this work no humans, no animals and no data is collected form social media platforms.

## Related research article

“None”.

## Declaration of competing interest

The authors declare that they have no known competing financial interests or personal relationships that could have appeared to influence the work reported in this paper.

## Data Availability

The data that has been used is confidential.

## References

[1] Debnath S., Muthu S.S., Gardetti M.A. (2016). Sustainable Fibres for Fashion Industry.

[2] Kanagaraj J., Senthilvelan T., Panda R., Kavitha S. (2015). Eco-friendly waste management strategies for greener environment towards sustainable development in leather industry: a comprehensive review. J. Clean. Prod..

[3] Zhang Q., Khan M.U., Lin X., Yi W., Lei H. (2020). Green-composites produced from waste residue in pulp and paper industry: a sustainable way to manage industrial wastes. J. Clean. Prod..

[4] Samanta K.K., Basak S., Chattopadhyay S.K., Muthu S.S. (2015). Environmental Implications of Recycling and Recycled Products.

[5] Nourbakhsh A., Ashori A. (2010). Wood plastic composites from agro-waste materials: analysis of mechanical properties. Bioresour. Technol..

[6] Vaisanen T., Haapala A., Lappalainen R., TOMPPO L. (2016). Utilization of agricultural and forest industry waste and residues in natural fiber-polymer composites: a review. Waste Manag..

[7] Schettini E., Santagata G., Malinconico M., Immirzi B., Scarascia Mugnozza G., Vox G. (2013). Recycled wastes of tomato and hemp fibres for biodegradable pots: physico-chemical characterization and field performance. Resour. Conserv. Recycl..

[8] shahid-Ul-Islam S.M., Mohammad F. (2013). Perspectives for natural product based agents derived from industrial plants in textile applications—a review. J. Clean. Prod..

[9] Sen T., Reddy H.J. (2011). Various industrial applications of hemp, kinaf, flax and ramie natural fibres. Int. J. Innov. Manag. Technol..

[10] Cao H., Wool R.P., Bonanno P., Dan Q., Kramer J., lipschitz S. (2014). Development and evaluation of apparel and footwear made from renewable bio-based materials. Int. J. Fash. Des. Technol. Educ..

[11] Mohd A.F. (2024). Materials Today: Proceedings.

[12] Lipsita S., Mohanty S. (2023). Effect of beeswax on the mechanical and barrier properties of biodegradable films. Polym. Plast. Technol. Eng..

[13] Singh R.K. (2022). Effect of particle size of diatomaceous earth on mechanical properties of PLA composites. Polymers.

[14] Mohanty Amar K., Misra Manjusri, Drzal Lawrence T. (2005).

[15] Akindapo J.O., Harrison A., Sanusi O.M. (2014). Evaluation of mechanical properties of coconut shell fibres as reinforcement material in epoxy matrix. Int. J. Engg. Res. Technol..

[16] Rampur Vinod V., Banagar Ashok R., Ganesh U.L., Srinivas C.V., Reur S.C. (2020). AIP Conference Proceedings 2247.

[17] Lu X., Gu X. (2023). A review on lignin-based epoxy resins: lignin effects on their synthesis and properties. Int. J. Biol. Macromol..

[18] Bagheri S., Nejad M. (2023). Fully biobased composite made with epoxidized-lignin, reinforced with bamboo fibers. Polym. Compos..

[19] Liu G., Jin C., Huo S., Kong Z., Chu F. (2021). Preparation and properties of novel bio-based epoxy resin thermosets from lignin oligomers and cardanol. Int. J. Biol. Macromol..

[20] Ferdosian F., Zhang Y., Yuan Z., Anderson M., Xu C.C. (2016). Curing kinetics and mechanical properties of bio-based epoxy composites comprising lignin-based epoxy resins. Eur. Polym. J..

[21] Limwibul Varuj, Jongvivatsakul Pitcha, Jirawattanasomkul Tidarut, Likitlersuang Suched, Dai Jian-Guo (2025). Bioresin-based composites reinforced with natural fibers and carbon fiber: mechanical properties and sustainable benefit assessment. J. Mater. Res. Technol..

[22] Naik Nithesh, Sooriyaperakasam Nilakshman, Abeykoon Yashoda K., Wijayarathna Yomali S., Pranesh G., Roy Soumik, Negi Rovin, Aakif Budnar Kunjibettu, Kulatunga Asela, Kandasamy Jayakrishna (2022). Sustainable Green composites: a review of mechanical characterization, morphological studies, chemical treatments, and their processing methods. J. Comput. Mech. Manag..

[23] Capretti M., Giammaria V., Santulli C., Boria S., Del Bianco G. (2023). Use of bio-epoxies and their effect on the performance of polymer composites: a critical review. Polymers.

[24] Woreta H.B., Bogale T.M. (2025). Mechanical and physical properties characterization of hybrid aloe vera and raffia palm fibers reinforced polyester composite material. J. Eng. Fiber. Fabr..

[25] Wang Y., Jin B., Ye D., Liu Z. (2022). Fully recyclable carbon fiber reinforced vanillin-based epoxy vitrimers. Eur. Polym. J..

[26] Nabipour H., Niu H., Wang X., Batool S., Hu Y. (2021). Fully bio-based epoxy resin derived from vanillin with flame retardancy and degradability. React. Funct. Polym..

[27] Mattar N., de Anda A.R., Vahabi H., Renard E., Langlois V. (2020). Resorcinol-based epoxy resins hardened with limonene and eugenol derivatives: from the synthesis of renewable diamines to the mechanical properties of biobased thermosets. ACS Sustain. Chem. Eng..

[28] Mattar N., Langlois V., Renard E., Rademacker T., Hübner F., Demleitner M., Altstädt V., Ruckdäschel H. (2021). Fully bio-based epoxy-amine thermosets reinforced with recycled carbon fibers as a low carbon-footprint composite alternative. Appl. Polym. Mater..

[29] Desai P.D., Jagtap R.N. (2021). Synthesis and characterization of Fiber-reinforced resorcinol epoxy acrylate applied to stereolithography 3D printing. ACS. Omega.

[30] Kim Y.O., Cho J., Kim Y.N., Kim K.W., Lee B.W., Kim J.W., Kim M., Jung Y.C. (2020). Recyclable, flame-retardant and smokesuppressing tannic acid-based carbon-fiber-reinforced plastic. Compos. B Eng..

[31] Shibata M., Nakai K. (2010). Preparation and properties of biocomposites composed of bio-based epoxy resin, tannic acid, and microfibrillated cellulose. J. Polym. Sci. B Polym. Phys..

[32] Borah N., Karak N. (2021). Tannic acid based bio-based epoxy thermosets: evaluation of thermal, mechanical, and biodegradable behaviors. J. Appl. Polym. Sci..

[33] Duraccio D., Di Maro M., Vaccaro F., Faga M.G., Bartoli M., Auriemma F., Milazzo M., Ruiz de Ballesteros O., Petrozziello M., Carpignano A., Gerboni R., Malucelli G., Asproudi A. (2025). Improving wear resistance of epoxy resin using bio-oil from hemp biomass: a sound strategy to reduce environmental impact. J. Clean. Prod..

[34] Latif Farah Ezzah A., Abidin Zurina Zainal, Cardona Francisco, Biak Dayang R.Awang, Abdan Khalina, Tahir Paridah Mohd, Ern Liew Kan (2020). Bio-resin production through ethylene unsaturated carbon using vegetable oils. Processes.

[35] Jani S.P., Senthil Kumar A., Adam Khan &M. Uthaya Kumar M. (2016). Machinablity of hybrid natural Fiber composite with and without filler as reinforcement. Mater. Manuf. Process..

[36] Jani S.P., Senthil Kumar A., Adam KhanORCID Icon M., Sajith &A. Saravanan S. (2021). Influence of natural filler on mechanical properties of hemp/Kevlar hybrid green composite and analysis of change in material behavior using acoustic emission. J. Nat. Fibers.

[37] Jani S.P., Jose A.S., Rajaganapathy C. (2022). A polymer resin matrix modified by coconut filler and its effect on structural behavior of glass fiber-reinforced polymer composites. Iran Polym. J..

[38] Li Z., Bai Y., Zang J., Liu W., Zhang C., Liu M., Gao H., He Y. (2023). Preparation and properties of a beeswax/water pickering emulsion stabilized by TEMPO-oxidized cellulose nanofibers and glycerol. Nanomaterials.

[39] Wood, Chapter 13, EN380 Naval Materials Science and Engineering Course Notes, U.S. Naval Academy (https://www.usna.edu/NAOE/_files/documents/Courses/EN380/Course_Notes/Ch13_Wood.pdf).

[40] Zhao S., Zhao J.X., Han G.Z. (2016). IOP Conf. Series: Materials Science and Engineering.

[41] Cai Z., Ross R.J. (2010). Wood handbook : wood as an engineering material: chapter 12. Centennial ed. General technical report FPL ; GTR-190.

